# The method for integrating dual-color fluorescence colocalization and single molecule photobleaching technology on the theophylline sensing platform

**DOI:** 10.1016/j.mex.2020.101155

**Published:** 2020-11-20

**Authors:** Dong Zhang, Shi Gang Liu, Zhaodi Fu, Yu He, Wenli Gao, Xingbo Shi

**Affiliations:** a Laboratory of Micro and Nano Biosensing Technology in Food Safety, Hunan Provincial Key Laboratory of Food Science and Biotechnology, College of Food Science and Technology, Hunan Agricultural University, Changsha 410128, China; b Analytical Testing Laboratory, Changsha Research Institute of Mining and Metallurgy CO., LTD., Changsha 410012, China

**Keywords:** Theophylline, Biosensing, Dual-color fluorescence colocalization, Single molecule photobleaching, Image J

## Abstract

• Smart usage of single molecule photobleaching technology and dual-color fluorescence colocalization is of critical importance for exploiting the sensing platform. Here, we provide the detailed protocols related to the article “A split aptamer sensing platform for highly sensitive detection of theophylline based on dual-color fluorescence colocalization and single molecule photobleaching” (published online by Biosensors and Bioelectronics) (Liu et al., 2020). The protocols contain: (1) how to clean the slides; (2) how to prepare the probe and detection sample; (3) Single molecule imaging; 4) Data processing by using the Image J. Finally, we used a simple model to confirm the feasibility of the method for integrating dual-color fluorescence colocalization and single molecule photobleaching technology on the theophylline sensing platform.

• A simple, ultrasensitive method for the detection of theophylline.

• The method is easily comprehensible.

• Both strategy formulation and data processing are simple, learnability, and highly reproducible.

## Graphical abstract

**Figure:**
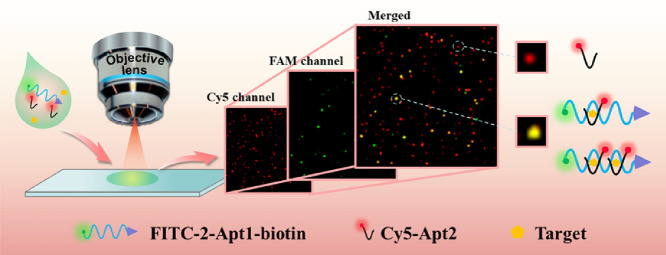

Specifications table

**Table:** 

Subject Area:	Chemistry
More specific subject area:	*Single molecule detection*
Method name:	*A split aptamer sensing strategy based on dual-color fluorescence colocalization and single molecule photobleaching technology*
Name and reference of original method:	*Liu* et al. *Biosensors and Bioelectronics,2020,166: 112,461* *Shi* et al. *Analytical Chemistry, 2018, 90(6) 3661–3665*
Resource availability:	https://imagej.nih.gov/ij/index.html

## Background

Single molecule photobleaching (SMPB) shows its promising potential to evaluate the degree of aggregation (DOA) at the molecule level, which is widely used for understanding protein aggregation, counting biological macromolecules in bio-machines and nanostructures [1], [2], [3], [4], and exploiting single molecule high-resolution method [5]. SMPB can count the number of fluorophores based on the stepwise photobleaching [6]. Under continuous illumination, the intensity time trace from an individual fluorescence spot shows consecutive photobleaching steps, and each step indicates one fluorescence molecule. Counting these photobleaching steps can provide the number of fluorescence molecules within a diffraction limited fluorescent spot.

In the field of ultrasensitive biosensor, it is also an effective way to improve the detection sensitivity with a couple of modified split aptamers which can provide sufficient recognition sites for fluorophores. In 2018, we developed a SMPB-based sensing strategy for the ultrasensitive detection of adenosine [7], in which the limit-of-detection (LOD) was down to 44.5 pM, which is the lower than the other earlier reported results. The specific binding of dye-labeled short strand DNA probes onto the elongated aptamer strand in the presence of adenosine resulted in a concentration-dependent self-aggregation process. The degree-of-aggregation (DOA) of the short DNA probes on the elongated aptamer strand could then be accurately determined based on the single molecule photobleaching measurement. This sensing strategy affords a universal sensing platform for detecting biomolecules. However, there are two limits. The one is that a large number of invalid data from unbound fluorophores was counted, and the false-positive results induced by the self-aggregation of fluorescent molecules cannot be identified. The other is by the requirement of long sequences of DNA/RNA aptamer in the design of recognition model.

The first limit can be conquered by dual-color fluorescence colocalization which is based on the simultaneous acquisition and analysis of the signal from two dyes [8], [9], [10]. If introducing one dye (Green fluorophore, FITC) serves as a counting reference and another dye (red fluorophore, Cy5) is utilized for quantitative counting of photobleaching steps, the false-positive result induced by the self-aggregation of fluorophores can be partly circumvented, and the detection efficiency is further improved.

RNA aptamers are playing important role in ultrasensitive biosensor, but the long RNA aptamers are more difficult to be synthesized than DNA aptamers because they are easily decomposed by nucleases [11], so RNA aptamers cannot provide sufficient recognition sites by themselves. Here we are introducing biotin-streptavidin linkage to overcome the second limit because each streptavidin has four biotin-binding sites. Briefly, a streptavidin-based probe (FITC-2-Apt1-SA) is formed by one streptavidin and four biotinylated RNA fragments labeled with fluorescein isothiocyanate (FITC-2-Apt1-biotin) via biotin-streptavidin linkage. Because each FITC-2-Apt1-biotin contains two repeating aptamer fragments, the FITC-2-Apt1-SA possesses up to eight binding sites of complementary aptamer fragments labeled with Cy5 dye (Cy5-Apt2).

Herein, we develop a new split aptamer-based sensing platform integrating SMPB technique and dual-color fluorescence colocalization. The following detailed protocol is about how to use this platform to test the model molecule-theophylline.

## Materials

1. Theophylline, caffeine, theobromine, guanosine, serine, and gallic acid (J&K Scientific Ltd. Beijing, China).
2. Hydrochloric acid, potassium dichromate,  sulfuric acid (Sinopharm Group Chemical Reagent Co., Ltd., Shanghai, China)
3. Anhua dark tea (AnHua Hunan, China).
4. Streptavidin, DEPC (diethyl pyrocarbonate) water, and 20X DPEC-treated PBS buffer (Shanghai Sangon Biotech Co., Ltd., Shanghai, China).
5. Immobilized streptavidin resin (Cat. # 786–390, A Geno Technology, Inc. USA).
6. Spin columns (1 mL, A Geno Technology, Inc. USA).
7. RNA probes (Shanghai Sangon Biotech Co., Ltd. Shanghai, China).
8. Ultrapure water with a resistivity of 18.2 MΩ.cm (Mingche D-24UV, Merck Millipore, MA, USA).
9. Image J software (National Institutes of Health, USA).

## Instrumentation

1. An epifluorescence microscope (BX51, Olympus, Japan) with 100X objective(NA = 1.45, UPLSAPO, Olympus, Japan) and electron multiplying charge coupled device (EMCCD, iXon3 DU-897D, Andor Technology, North Ireland) on an air floating isolated optical platform (ZLT18–12, Beijing Zolix Instrument Co., Ltd. Beijing, China).
2. The light source is a mercury lamp (100 w, USH-1030 L, Olympus, Japan). To investigate the photobleaching steps, a neutral-density filter ND25 (U-25ND25, Olympus, Japan) with its aperture diaphragm set at 1/2 was fixed in the light path to weaken the light intensity, and two filters of Cy5 (Semrock, American) and Fam (Semrock, American) can be switched into the light path for the required wavelength.

## Protocol

### Slide cleaning

**Fig. 1:**
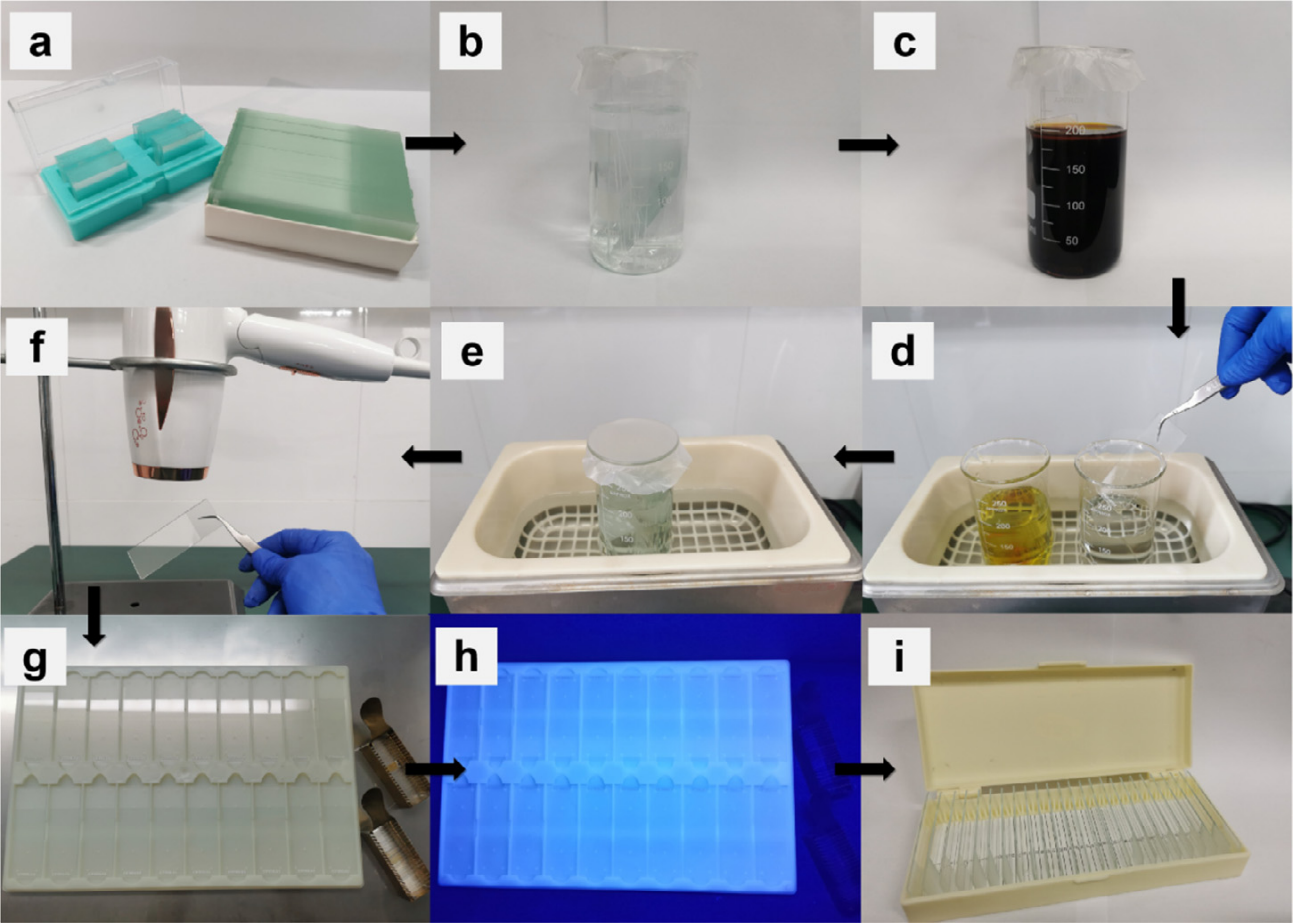
Steps for cleaning slides (a) Remove the slides from the box. (b) Clean glass slides and coverslips by 2% hydrochloric acid solution. (c) Clean glass slides and coverslips by potassium dichromate lotion. (d) and (e) Clean the slides in ultrapure water by ultrasonication. (f) and (g) Select and dry slides. (h) Bleach the slides by an ultraviolet lamp. (i) Store the slides.

The complete washing process of glass slides is shown in Fig. 1.

1. Remove the slides from the box. (see Fig. 1a)
2. Place glass slides and coverslips into 2% hydrochloric acid solution for 2 h and rinse them off the hydrochloric acid residue with tap water. (see Fig. 1b)
3. Put them into potassium dichromate lotion overnight and take them out, then wash them with tap water one by one. (see Fig. 1c& 1d)
4. Ultrasonically clean the slides in ultrapure water for 5 min to remove the impurity and repeat it three times. (see Fig. 1e)
5. Select the slides with smooth surfaces and no scars after cleaning. (see Fig. 1f)
6. Dry the selected slides by an electric hair dryer one by one. (see Fig. 1g)
7. Irradiate the dried slides with an ultraviolet lamp for 2 h to bleach the possible fluorescent substances. (see Fig. 1h)
8. Put them in a dry crystalline slide box treated with DPEC to prevent contamination from exogenous RNase and prepare for the next step of sample preparation. (see Fig. 1i)

**Notes1:** Cover the container mouth with a sealing film to avoid dust during soaking the glass slides.

**Notes2:** It is best to use the cleaning slides in time to avoid contamination by floating particles in the air. These cleaned slides that have been stored for a long time should be washed again before use.

**Notes3:** To avoid RNase contamination, all glassware should be dried for 15 min or longer at 180 °C before use; all plastic utensils are soaked into 0.1% DEPC water or rinsed with chloroform; all operation processing should be performed in a special laboratory for RNA operation; and the operator wears disposable masks, hats, and gloves. The gloves should be changed frequently during the experiment.

### Probe design and validation

1. Probe design: The design of two ribonucleotide fragments is modified on the previous report as a self-assembling RNA aptamer to detect the theophylline [12]. The same split site on the loop region which hardly affects the binding properties is taken, but one sequence of the aptamer is repeated twice, labeled with FITC at the 5′ end and biotin at the 3′ end, respectively (FITC-2-Apt1-biotin). The Apt2 is the other complementary fragment of the split theophylline aptamers and labeled with Cy5 at the 5′ end (Cy5-Apt2). In the presence of theophylline, the FITC-2-Apt1-biotin probe can bind two Cy5-Apt2 probes. The sequences and modifications are as follows:
  FITC-2-Apt1-biotin: 5′-FITC-rGrGrCrCrCrUrUrGrGrCrArGrCrGrUrCrGrGrCrCrCrUrUrGrGrCrArGrCrGrUrC-biotin-3′.
  Cy5-Apt2: 5′- Cy5-rGrGrCrGrArUrArCrCrArGrCrCrGrAArA −3′
2. RNA probes were synthesized by a commercial company. (Shanghai Sangon Biotech Co., Ltd. Shanghai, China)
3. Probe validation: The photobleaching step number is just one in the absence of theophylline because the Cy5-Apt2 is free. In the presence of theophylline, the photobleaching step number will be counted to be higher because the Cy5-Apt2 is bound to FITC-2-Apt1-SA due to the strong interaction between aptamer and theophylline.

### Probe preparation

1. Activation of RNA strands

Take out the RNA strands in −20 °C refrigerator. Centrifuge at 4000 rpm for 1 min to centrifuge the lyophilized RNA powder to the bottom of the tube wall. Slowly open the tube cap, add some DPEC water, and then close the tube cap, shake to dissolve.

**Notes4:** Unused RNA should be stored below −20 °C. The liquid dosage dissolving from RNA is recommended to store it individually to avoid multiple freeze-thaw affecting the RNA strands activity.

2. FITC −2-Apt1-SA preparation

**Fig. 2:**
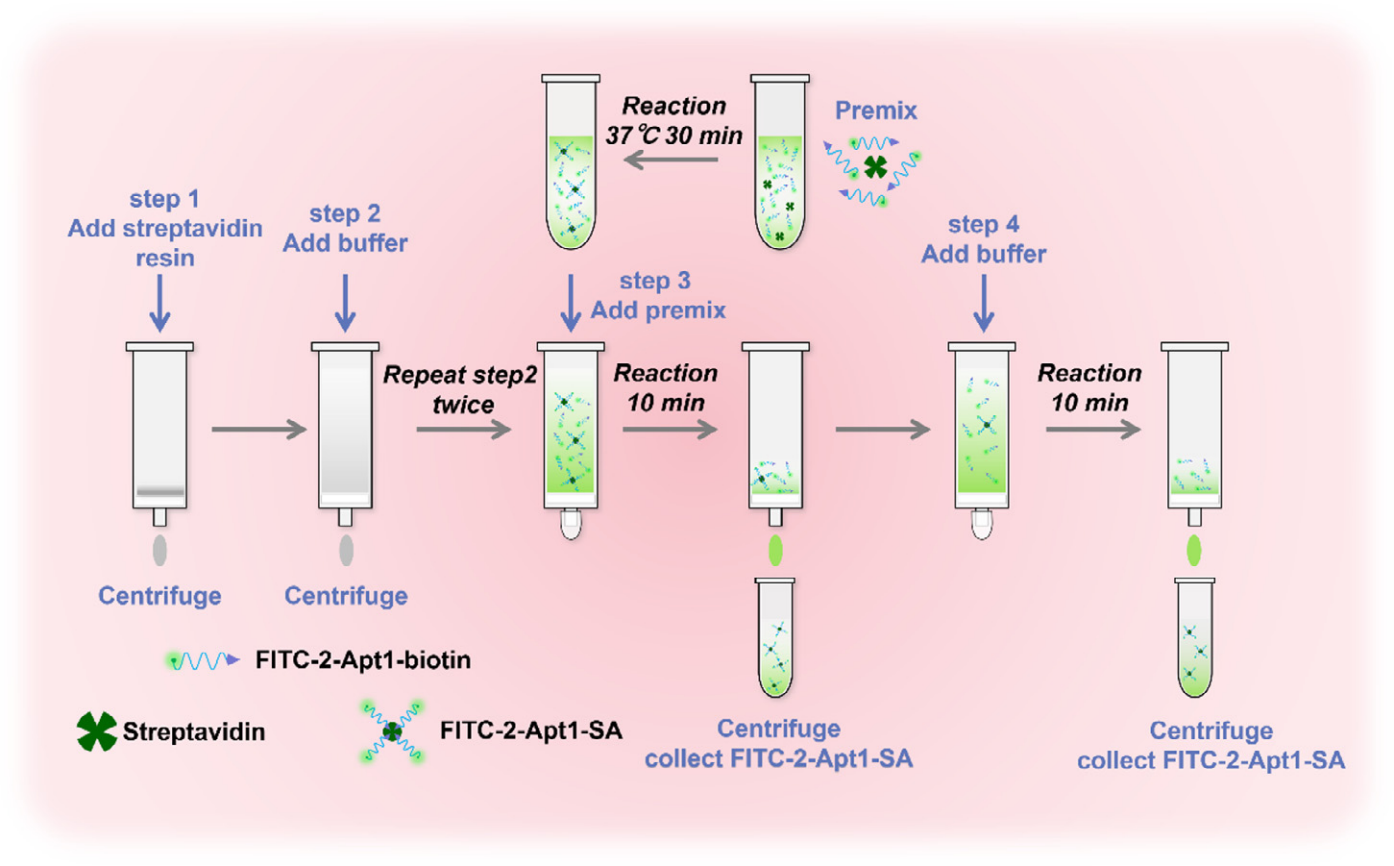
Schematic diagram of FITC-2-Apt-SA probe preparation.

The streptavidin and FITC-2-Apt1-biotin are premixed at a molar ratio of 1:4 and kept at 37 °C for 30 min. The FITC-2-Apt2-SA complex can be prepared following the protocol which is shown in Fig. 2.

(1) Equilibrate the resin and reagents to room temperature, and pack 10 µL of streptavidin resin into the column. Then centrifuge at 500 g for 1 min to remove the buffer. (see step 1 in Fig. 2)
(2) Add 1 mL of buffer and centrifuge at 500 *g* for 1 min and repeat step 2 twice. (see step 2 in Fig. 2)
(3) Place the column in a new collection tube and add the premix solution (125 µL, 20 µM) to the column. Centrifuge the blocked column at 500 *g* for 1 min after incubating it at room temperature for 10 min, and collect fractions. (see step 3 in Fig. 2)
(4) Add 1 mL of buffer to wash the column and collect fractions again. (see step 4 in Fig. 2) Repeat step 4.
(5) Finally, mix the three centrifugation fractions and volume it up to 3 mL.
(6) The concentration of FITC-2-Apt1-SA acquisition can be monitored by the absorbance at 280 nm.

3. Preparation of the detection solution

Cy5-Apt2 and FITC-2-Apt1-SA complex are mixed at a molar ratio of 8:1 using DPEC-treated PBS (0.01 M pH=7.4) buffer to prepare the theophylline detection solution.

### Sample preparation

1. Tea soup preparation

(1) Weigh 5 g dried tea precisely.
(2) Put it into a 300 mL triangulated bottle.
(3) Add 100 mL distilled water and boil it, and keep it in a boiling state for 15 min.
(4) Treat it with a vacuum pump for filtering, and extract the filter residue with 50 mL boiling water by twice.
(5) Combine the filtration with a constant volume of 200 mL.
(6) Dilute 100 times with DPEC water and sealed at 30 °C for later use.

2. The detection of sample preparation

(1) The probe solution is diluted at 8 nM for Cy5-Apt2 and 1 nM for FITC-2-Apt1-SA, respectively. The targets (theophylline and control sample) and the probe solution are mixed and reacted at a volume ratio of 1:2 at 25 °C for 0.5 h, and the final concentration of theophylline or other control samples in the reaction solution was controlled in the range of 1~8 nM.
(2) 4 µL of the reaction solution is put on the surface of a glass slide, and a coverslip is slowly covered along the edge of the droplet. To avoid the sample evaporation, coat evenly the coverslip with nail polish to seal the cavity between the cover glass and the slide. The prepared samples are incubated for another 10 min in a 37 °C water bath and then observed under a microscope.

### Single molecule imaging

(1) The prepared slides are mounted on the epifluorescence microscope.
(2) Switch filter assembly to Fam channel. The light source provided by the mercury lamp passes through an ND25 filter with its aperture diaphragm set at 1/2 and then the FITC filter to obtain the excitation light of the required wavelength. Once the sample is excited, the fluorescent photons pass through the 100X objective lens and are collected by the EMCCD working at −75 °C because imaging at low temperature can improve the capturing sensitivity and stability of signal capture. The imaging parameters are set as follows: the exposure time is 0.1–0.3 s; kinetic cycle time (the interval of consecutive frames) is 0.2–0.5 s, and the electron multiplying gain level is 200–500. The number of continuous frames in a photo is taken at 100.
(3) Switch filter assembly to Cy5 channel. The number of continuous frames in a photo is taken from 500 to 800 according to the requirement without changing the imaging field of view and EMCCD imaging parameters.
(4) To accurately obtain the photobleaching step numbers of fluorescent dyes, at least 500 consecutive frames should be acquired. And for each value of statistically averaged photobleaching steps, at least 200 merged fluorescent spots which are yellow in the merged spots should be observed, and all data should be obtained in triplicate.

**Notes5**: The exposure time and kinetic cycle time are important factors to capture the number of bleaching steps. When the light intensity is constant, the shorter the exposure time and kinetic cycle time is, the easier it is to capture the bleaching process. The DOA increases with increasing the number of targets, so the exposure time and the interval of consecutive frames would be adjusted appropriately without affecting the calculation of DOA.

**Notes6**: With a fixed aperture diaphragm, field diaphragm, and light intensity, the time for single-molecule imaging to completely capture the bleaching process is determined by the number of targets in the system. With no target, most of Cy5 dyes are bleached by one-step, and the bleaching time is fast. However, with the increasing amount of targets, the imaging time (kinetic cycle length) would be longer because the number of bleaching steps will be increased from one to eight and the aggregates are more resistant to bleaching during this process.

**Notes7:** The concentration of dyes should be nanomole level so that each fluorescent spot in the fluorescent image is more easily separated.

**Notes8:** The wavelengths of the two filters are different, resulting in a slight difference in the imaging focal plane, which needs to be adjusted manually.

**Notes9:** The use of precision optical platforms to reduce machine shaking and imaging in the darkroom is more conducive to signal capture.

### Data processing by using image J

**Fig. 3:**
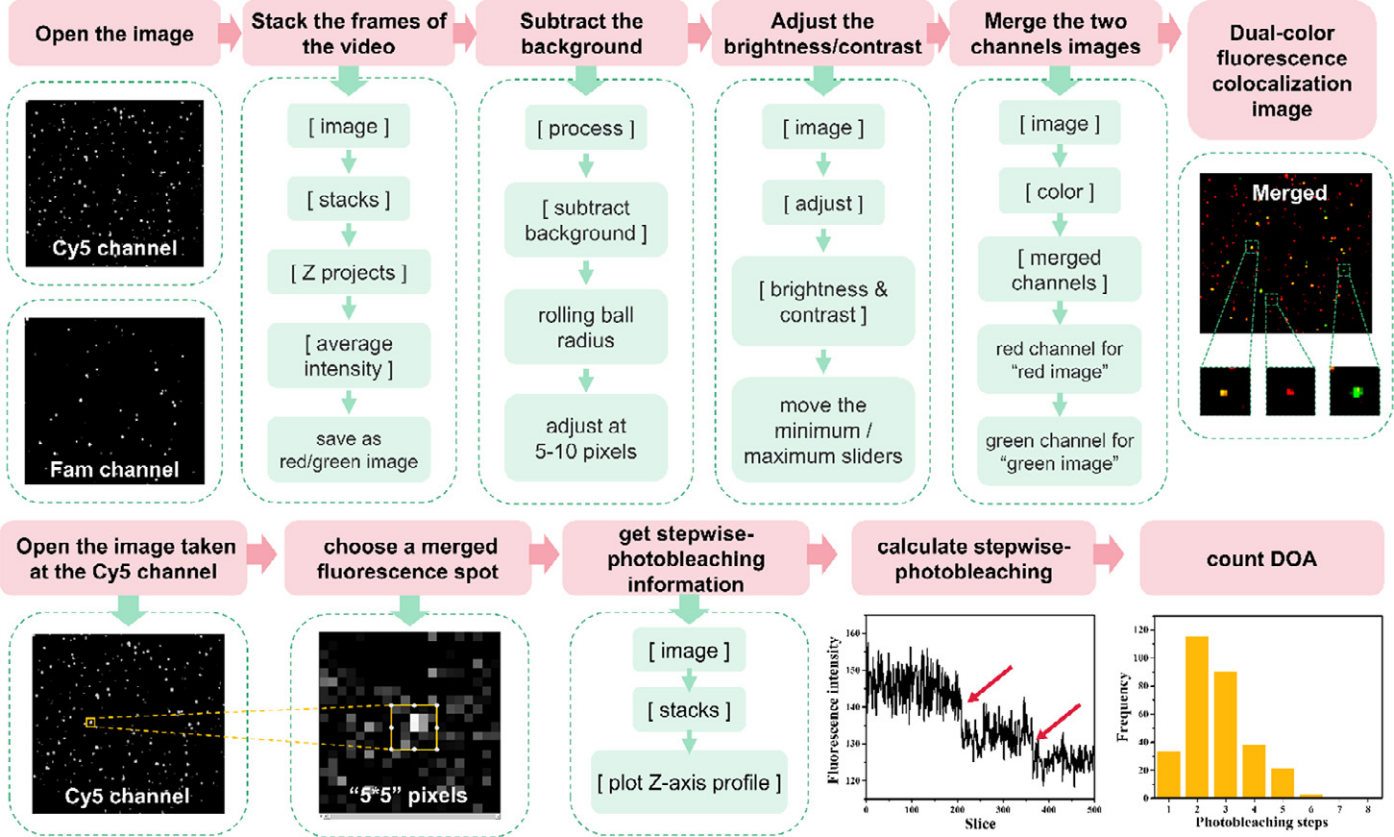
Data processing by using IMAGE J. The top of the processing is dual-color fluorescence colocalization image and the bottom of processing is the extracting the photobleaching data from the images. Inside “[]” is the menu button for clicking the software.

The complete data processing by using Image J is shown in Fig. 3.

1. Dual-color fluorescence colocalization

(1) Open the image. Click the “file” menu, and choose the target image (Cy5 channel image); or pull the target image into the software.
(2) Stack the frames of the video. Click the “image” toolbar and enter into the pull-down menu, choose “stacks” → “Z projects” and choose the “average intensity” in the appearance of the dialog window. Click the “OK” button, and a new AVG image is formed, and saved this new AVG image as “red image”.
(3) Subtract the background of the AVG image. Click the “process” toolbar and enter into the pull-down menu, choose “subtract background” and set the “rolling ball radius” at 5–10 pixels in the new dialog window.
(4) Adjust the brightness/contrast. Click the “image” toolbar and enter into the pull-down menu, choose “adjust” and open a new dialog window. According to the intensity of images, move the minimum or maximum sliders, until the black background appears and the signal spots light up. Finally, click the “apply” button.
(5) Repeat the 1–4 steps for the Fam channel image. The new AVG image is saved as “green image”.
(6) Merge the two channels images. Click the “image” toolbar and enter into the pull-down menu, choose “color” → “merged channels”. And then choose the “red channel” for “red image”, “green channel” for “green image”. Finally, click the “OK” button to obtain the “Dual-color fluorescence colocalization” image.

2. Extracting the photobleaching data from the images

(1) Use “rectangular” toolbar to choose the one fluorescence spot, the area is “5 × 5” pixels.

(2) Click the “image” toolbar and enter into the pull-down menu, choose “stacks” → “plot Z-axis profile” and stepwise- photobleaching information is shown at the pop-up window.

(3) Check the number of stepwise-photobleaching and verify the number of dyes in one fluorescence spot.

**Fig. 4:**
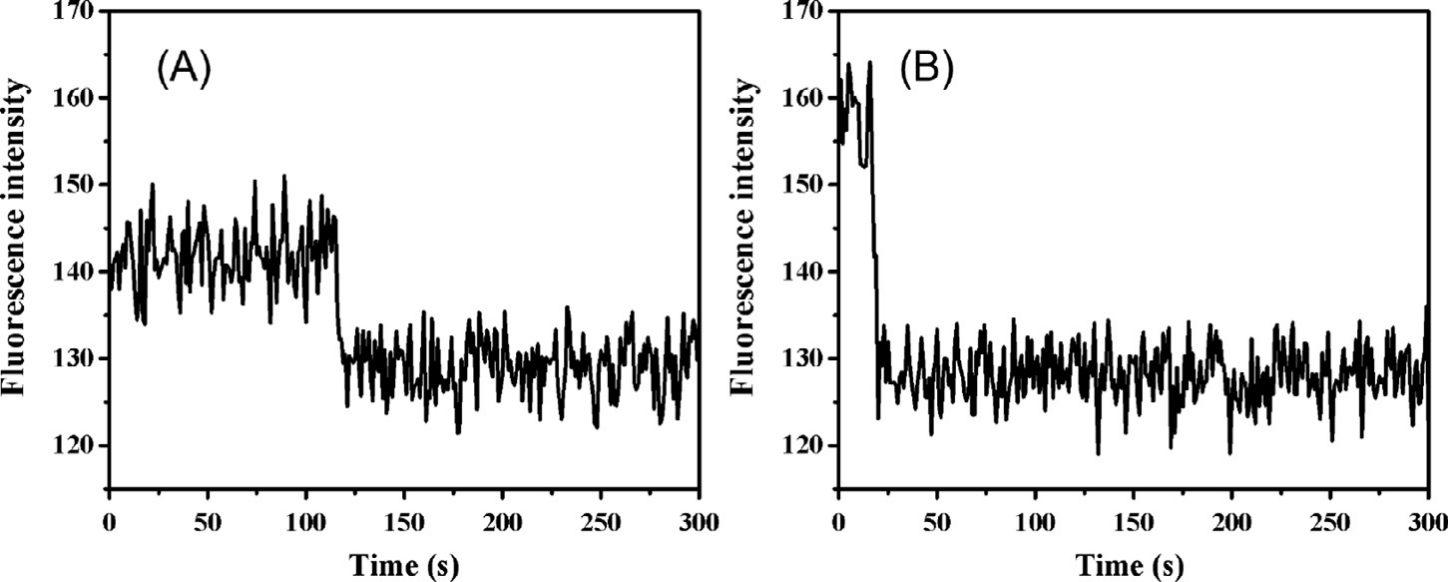
The degree of reduced fluorescence intensity assisting DOA calculation. (A) shows single-step photobleaching and indicates one bing dye in the fluorescence spot. (B) shows the bleaching steps from two binding dyes although it looks like a single-step-photobleaching because the decreased fluorescence intensity is two times of one binding dye molecule.

**Notes10:** Some steps may contain two or more dyes, in this situation, the degree of reduced fluorescence intensity can be used to assist the verification of the DOA calculation, as shown in Fig. 4.

## Method validation

**Fig. 5:**
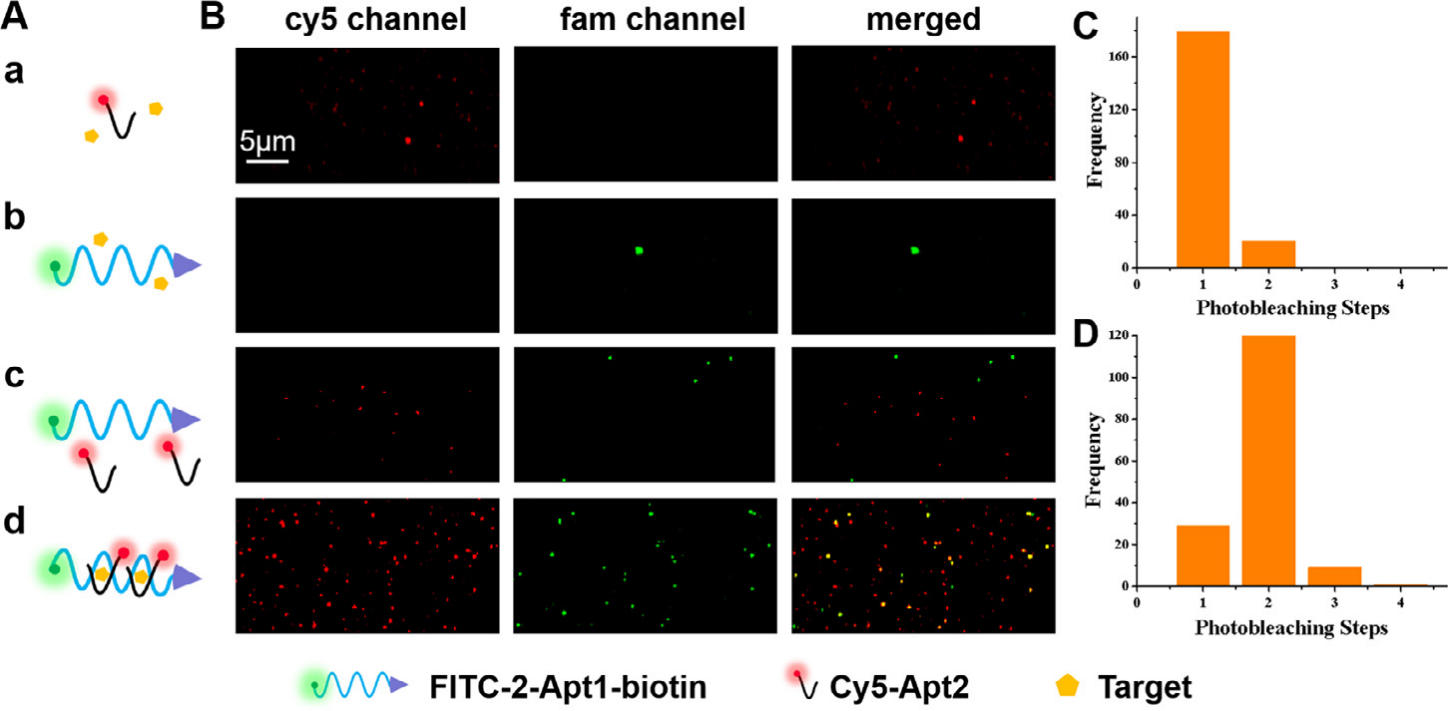
Schematic diagram of theophylline detection method. (A) Diagram of different probe solutions. Cy5-Apt2 added with theophylline (a), FITC-2-Apt1-biotin added with theophylline (b), the mixture of Cy5-Apt2 and FITC-2-Apt1-biotin without theophylline (c), the mixture of Cy5-Apt2 and FITC-2-Apt1-biotin added with theophylline (d). (B) The corresponding fluorescence imaging photographs from different channels. (C-D) Distribution histograms of statistically photobleaching steps of Cy5 in a and b solution.

In this assay, the concentration of FITC-2-Apt1-biotin and Cy5-Apt2 probe was kept at 1:2 with molar ratio, the concentration of theophylline was the same as that of FITC-2-Apt1. Fig. 5B shows fluorescence imaging photographs of Cy5-Apt2 added with theophylline (a), FITC-2-Apt1-biotin added with theophylline (b), the mixture of Cy5-Apt2 and FITC-2-Apt1-biotin without theophylline (c), the mixture of Cy5-Apt 2 and FITC-2-Apt1-biotin added with theophylline (d). It can be found only the sample for the mixture of Cy5-Apt2 and FITC-2-Apt1-biotin added with theophylline shows deep yellow fluorescent spots in the merged images (Fig. 5B bottom line). The distribution histograms of statistically photobleaching steps of Cy5 in pure Cy5-Apt2 added the solution with theophylline and the mixture of Cy5-Apt2 and FITC-2-Apt1-biotin added with theophylline are exhibited in Fig. 5C-D. It can be observed that the mixture of Cy5-Apt2 and FITC-2-Apt1-biotin added with theophylline shows largely 2 steps. These experimental results indicate that the proposed strategy is highly feasible for theophylline sensing. In addition, these results combining with the results of the published article [1] indicate the sensing platform integrated with dual-color fluorescence colocalization and single molecule photobleaching technology possesses potential utility for highly sensitive detection of theophylline in real samples even that there are many food substrates.

## Declaration of Competing Interest

The authors declare that they have no known competing financial interests or personal relationships that could have appeared to influence the work reported in this paper.
